# Effects of occupational balance on subjective health, quality of life, and health-related variables in community-dwelling older adults: A structural equation modeling approach

**DOI:** 10.1371/journal.pone.0246887

**Published:** 2021-02-11

**Authors:** Sangmi Park, Hae Jong Lee, Byoung-Jin Jeon, Eun-Young Yoo, Jong-Bae Kim, Ji-Hyuk Park

**Affiliations:** 1 Department of Occupation Therapy, Yonsei University, Wonju, Republic of Korea; 2 Department of Health Administration, Yonsei University Wonju, Wonju, Republic of Korea; 3 Department of Occupational Therapy, Kangwon National University, Samcheok, Republic of Korea; Universiti Sains Malaysia, MALAYSIA

## Abstract

Occupational balance is an important variable associated with health and quality of life. This study aimed to investigate the influence of occupational balance on health, quality of life, and other health-related variables using structural equation modeling. We analyzed data from 208 adults over 55 years old. Mean age of the participants was 70.21 years (SD 7.22). The research model for analysis was based on the results of previous studies addressing occupational balance and related variables such as stress, leisure satisfaction, life satisfaction, subjective health, quality of life, and participation. General fit indices of the final model were acceptable (x^2^/df = 1.708, *p* < .001, RMSEA = .058, TLI = .923, CFI = .929, and SRMR = .067). Although the size of effect was small to medium (.157–.249), occupational balance was identified as an independent variable directly or indirectly affecting subjective health, quality of life, and health-related variables in the final model. Our results showed that it is possible to improve subjective health and quality of life by promoting better occupational balance. Further studies developing an intervention program based on occupational balance are required to confirm the feasibility of the intervention and its effect on older adults’ health and quality of life in real-life circumstances.

## Introduction

In occupational therapy, occupation refers to meaningful activities that individuals engage in [1]. The areas of occupation include entire activities of human life [1]. Balance between occupations is positively related to personal health, happiness, and wellbeing [1, 2]. However, occupational imbalance may cause disease and unhappiness [1]. Occupational imbalance means stress or boredom due to an inappropriate level of occupational engagement [3]. There is a negative association between wellbeing and occupational imbalance [3].

We do not have a unanimous definition of occupational balance [1]; nevertheless, a few different definitions exist. Wilcock and Hocking [4] define occupational balance as balance on occupational engagement that leads to wellbeing. Occupational balance can also be defined as a subjective insight of having a proper level of occupation in terms of diversity and quantity [5], or having quantitatively satisfactory daily occupational patterns [6]. Although there is no consensus on the definitions of occupational balance, we can identify a common feature regarding its concept, such as personal subjectivity and its relation to health, wellbeing, and satisfaction. The concept of life balance is similar to that of occupational balance [7]. In the life balance model [8], life balance is defined as a daily activity pattern that is healthy, meaningful, and sustainable in the present environment, so that it gives satisfaction.

Previous studies on occupational balance involved its measurement [7, 9], comparison between groups demonstrating different levels of occupational balance [2, 10–12], and association between occupational balance and other variables [2, 13].

As life cycles change, patterns of daily occupation also alter [14]. Individuals experience occupational transition when a life event such as the loss of a loved one or retirement occurs [14]. In this transitional period, it can be useful to apply the approaches of occupational balance [15]. Especially for older adults, balance between occupations can help create new daily routines while experiencing successful occupational transition [14, 15]. As older adults age, diversity in their daily activities tends to decrease, and they spend more time on passive leisure activities [16]. It is recommended for older adults to explore new activities to help them spend as much active time as possible [14, 15]. Daily routine without meaningful activities and with a lower level of activity can lead to a decline in the physical or cognitive functions in the older population [17]. In addition, maintaining participation in everyday activities is crucial for personal wellbeing and quality of life [18]. When individuals participate in diverse occupations, they can achieve a harmonious status in participation [19]. Partaking in diverse occupations is essential for individuals to have an occupationally balanced status [19]. For older adults aged above 65, achieving occupational balance has a positive effect on protecting their health status. Other benefits of achieving occupational balance comprise lower levels of stress and higher levels of wellbeing or health [2, 7].

Previous studies reported the importance of occupational balance as a health-related factor [2, 3, 12, 13], and suggested the possibility of using the concept of occupational balance for improving health [4, 20]. However, there are limited studies on the influence of occupational balance as an independent variable that can affect health or quality of life. The purpose of this study was to investigate the associations between occupational balance and related variables such as leisure, stress, life satisfaction, health, quality of life, and participation, and examine the effects of occupational balance on the aforementioned variables.

## Materials and methods

### Procedure

**Fig 1:**
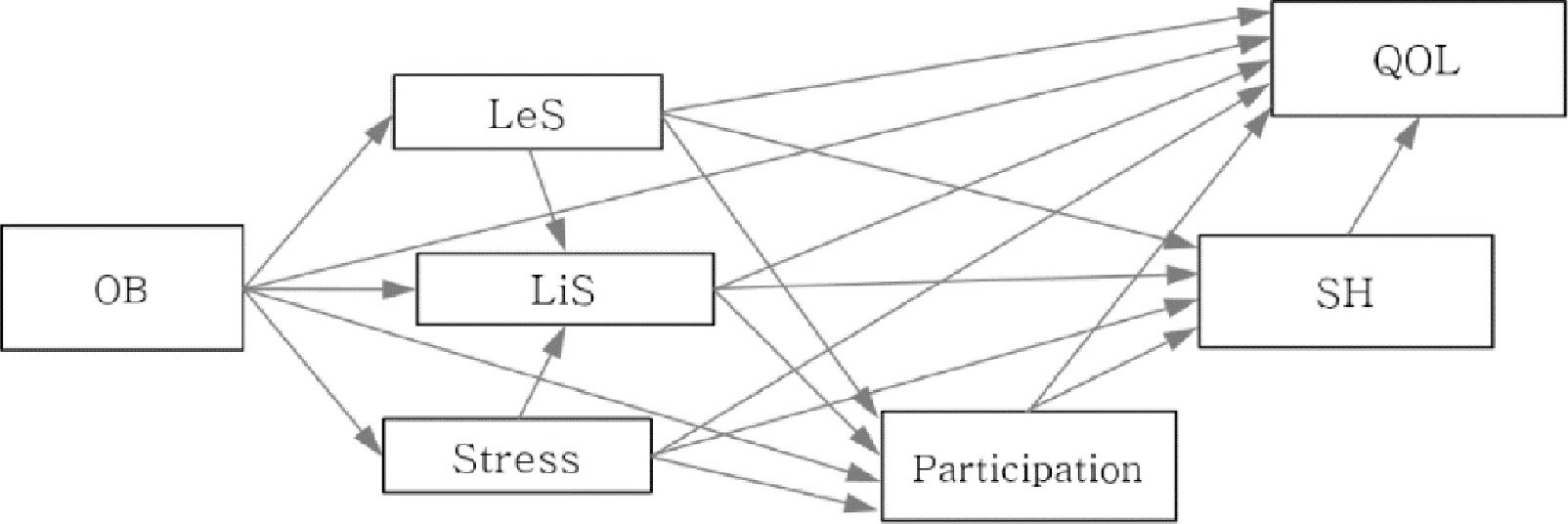
Conceptual framework. OB = occupational balance; LeS = leisure satisfaction; LiS = life satisfaction; SH = subjective health; QOL = quality of life.

The present cross-sectional study was conducted in three steps. First, we organized a literature review about occupational balance to set the framework of the research model (Fig 1). Second, we conducted a survey using a questionnaire packet comprising seven assessment tools. All measurements included in this study could be self-reported by participants. Third, we examined and modified the research model to improve both the fit indices and the statistical significance of the paths. After modifying the model, the total effect of the variables was analyzed.

### Research model

We used validated assessment tools to measure the seven latent variables. Subtotal or averaged scores of subcategories in each assessment tool were used as observed variables.

All paths were set based on the published literature. The variables of leisure satisfaction, life satisfaction, and stress were considered as the mediating factors between exogenous variables of occupational balance and endogenous variables of participation, subjective health, and quality of life [21–25]. We set the direct paths from occupational balance to quality of life based on the association between quality of life and satisfaction obtained by participating in daily occupations [1, 8].

Paths between variables were supported by the associations between leisure satisfaction and both life satisfaction and quality of life [1, 26], physical activities in leisure activities and both subjective health and participation [17, 24], and relationships between stress and life satisfaction, subjective health, and quality of life [27, 28].

Participation mediated by occupational balance [23] had an effect on both subjective health and quality of life [29, 30]. Subjective health affected the quality of life [31].

### Participants

This study was approved by the Institutional Review Board of Yonsei University Wonju Campus (1041849-201806-BM-050-01), and written informed consent was obtained from all included participants. The sample size was determined by the threshold for the statistical analysis. Collecting at least 200 cases was recommended to use maximum likelihood estimation of structural equation modeling [32]. A total of 225 community-dwelling older adults living in the metropolis and small- to medium-sized cities were included. After excluding five incomplete questionnaires and 12 outliers detected by Mahalanobis distance, a total of 208 sets of responses were analyzed.

Inclusion criteria were adults aged 55 years or above, who were living independently in their homes for at least one year. Convenience and snowball sampling were used. We recruited most of the participants from two community centers, one located in a metropolis and the other in a medium-sized city. The respondents participated in this study voluntarily after they read the information in flyers posted in their community centers. We set desks for the participants to answer the questionnaire, and there was at least one researcher to help the participants when they had a question about the research or how to answer the questionnaires. We collected the data through interviews when the participants who visited desks wanted the interview method instead of self-reporting. This was done to minimize the response errors that could have been caused by misunderstanding on how to answer the questions. We also adopted snowball sampling method mainly for participants under 65 years old. As the majority of the community center users were retired older adults aged 65 or more, we could collect data from the younger participants through snowball sampling. Snowball sampling was conducted by asking participants who completed the questionnaires to deliver an extra questionnaire packet to their acquaintances who met the inclusion criteria. It took approximately 45 minutes on an average to answer the questionnaire packet. Every participant completed the questionnaire and received the compensation corresponding to minimum wage for an hour.

### Measurements

#### Korean version of Life Balance Inventory (K-LBI)

The Korean version of the Life Balance Inventory (K-LBI) [33] was used to measure occupational balance. In a validation study, a significant correlation of the K-LBI with the Korean version of the WHOQOL-BREF was reported [33]. Average item-level content validity indices of 53 items in the K-LBI was 0.79 [33]. The Life Balance Inventory (LBI) [7], which is an original version of the K-LBI, is a self-reported assessment tool for occupational balance [1]. It contains 53 daily activities such as getting adequate sleep, working for pay, cooking, or taking care of pets, and these items are categorized into the following four subcategories: health, identity, relationship, and challenge and interest. Participants self-rate the congruence about time use by checking whether they satisfactorily spent time on each activity during the last four weeks. The score for each item ranges from 1 (always more or less than I want) to 3 (almost or as much as I want). The respondents may skip scoring of the activities that they did not participate in or had no interest of performing during the last four weeks. The average score of all scored activities is used to assess participants’ level of general occupational balance. When the scores are closer to 3, the respondent is considered to have a higher level of occupational balance. In addition, it is possible to compare the scores of the four subcategories. Cronbach’s α for LBI and K-LBI were .89–.97 [7] and .83–.88 [33], respectively. The reliability in this study was Cronbach’s α = .875.

#### Korean versions of the World Health Organization Disability Assessment Schedule 2.0 (KWHODAS 2.0)

Twelve items in the Korean versions of the World Health Organization Disability Assessment Schedule 2.0 (KWHODAS 2.0) [34] were used to measure subjective health. The KWHODAS 2.0 contains 12 items in six subcategories: cognition, mobility, self-care, getting along, life activities, and participation. The scores of the six subcategories in KWHODAS 2.0 reflect how much functional limitation the individual perceived in his or her daily activities [34]. Concurrent validity of the KWHODAS was confirmed with the high level of correlation (*r* = .77) between the KWHODAS and the Korean Functional Rating Index [35]. The test-retest reliability was intra-class correlation coefficient (ICC) = .94 (self-rated score), and the inter-scorer reliability was ICC = .94–1.00 (interviewer rated score) [34].

In this study, we used raw scores measured on a five-point-Likert scale (1: extreme difficulty or cannot do, 2: severe difficulty, 3: moderate difficulty, 4: mild difficulty, 5: no difficulty at all) instead of computation scores. We interpreted a higher score as higher levels of subjective health. The reliability in this study was Cronbach’s α = .923.

#### Korean version of the WHO Quality of Life-Brief (WHOQOL-BREF)

Twenty five items in the Korean version of the WHO Quality of Life-Brief (WHOQOL) [36] were used to measure quality of life. Originally, the WHOQOL-BREF [36] contained 26 items that consisted of one question for general quality of life, one question for overall health, and 24 items subdivided into four subcategories: physical, psychological, social, and environmental quality of life. In this study, except one question for overall health, 25 items measured on a five-point-Likert scale were used for analysis. A higher score was interpreted as higher levels of quality of life. The reliability was Cronbach’s α = .898 [36]. The reliability in this study was Cronbach’s α = .937.

#### Korean Utrecht Scale for Evaluation of Rehabilitation-Participation (K-USER-P)

The Korean Utrecht Scale for Evaluation of Rehabilitation-Participation (K-USER-P) [37] was used to measure perception of participation in daily activities. K-USER-P is a validated Korean version of the Utrecht Scale for Evaluation of Rehabilitation-Participation (USER-P) [38]. The K-USER-P contains 32 items in three subcategories: frequency, restriction, and satisfaction. Higher scores in each subcategory indicated a higher level of engagement, lower restriction, and a higher level of satisfaction with participation. The reliability of the three subcategories for USER-P was Cronbach’s α = .70–.91 [38], and for K-USER-P was ICC = .66–.69 [37].

Originally, the K-USER-P was developed to assess participation for people with limitations in physical function. Although our sample was composed of relatively healthy older adults, we used the K-USER-P as a measurement for participation in this study for the following reasons: first, the K-USER-P was the only Korean assessment tool for measuring participation, and had been previously verified for its psychometric properties; and second, older adults can be considered a population with latent limitations in physical function. The reliability in this study was Cronbach’s α = .703.

#### Leisure Satisfaction Scale (LSS)

The Leisure Satisfaction Scale (LSS) was originally developed by Beard and Regheb [39], and translated into Korean and validated by Kim, Lee, and Hwang [26]. The scale contains 24 items in six subcategories: psychological, relaxational, aesthetic, social, physiological, and educational factors. The content validity of 24 items in the LSS was examined with a review process by professionals in leisure studies, and the six subcategories were verified through explanatory and confirmatory factor analyses [26]. Higher scores indicated higher levels of leisure satisfaction. The reliability in this study was Cronbach’s α = .958.

#### Korean version of Perceived Stress Scale (K-PSS)

The Korean version of the Perceived Stress Scale **(**K-PSS) was originally developed by Cohen, Kamarck, and Mermelstein [38], and translated into Korean and validated by Park and Seo [40]. The scale comprises five positive and five negative types of questions. In this study, we only used the five negative items for analysis because of the low factor loading of positive items on stress. Higher scores indicated higher levels of stress. The reliability was Cronbach’s α = .85 [40]. The reliability in this study was Cronbach’s α = .849.

#### Korean version of the Satisfaction with the Life Scale (K-SWLS)

Five items in the Korean version of the Satisfaction with the Life Scale (K-SWLS) [41] were used to assess life satisfaction and measured on a five-point-Likert scale. The K-SWLS was originally developed by Diener, Emmons, Larsen, and Griffin [42], and translated into Korean and validated by Cho and Cha [41]. Items in the SWLS [42] were validated by diverse age groups in many countries, and widely used to assess individual life satisfaction [43]. We adopted a five-point Likert scale in this study instead of the original seven-point Likert scale to alleviate any confusion that might be caused by the different scale, since most of questions in the questionnaire packet were measured on five-point Likert scales. Higher scores indicated higher levels of satisfaction in life. Internal consistency was .85–.90 [41]. The reliability in this study was Cronbach’s α = .879.

### Statistical analysis

We used raw data collected through the survey for the analysis. Descriptive analysis was performed using the Statistical Package for the Social Sciences (SPSS) version 25, and structural examination, path analysis, and effect analysis were performed using Amos version 22.

Maximum likelihood was used to estimate the coefficient of direct path. Nonparametric bootstrapping using maximum likelihood was used to examine the significance of effectiveness through indirect path. Repetition of 500 times and 95% confidence interval were set.

Several model fit indices should be considered together to appropriately interpret the model. As we followed the recommendations of Boomsma [44] and McDonald and Ho [45], five indices including x^2^, *p* value, root mean square error of approximation (RMSEA), standard root mean square residual (SRMR), Tucker–Lewis index (TLI), and comparative fit index (CFI) were reported in this study.

RMSEA below .05 was considered a good model with few errors, .05–.08 as an acceptable model, and over 1 as an inappropriate model [46]. SRMR below .10 was considered acceptable [47].

When TLI and CFI were over .9, these were considered good indices [48].

The appropriate range of normed chi-square (NC) was between 2.0 and 3.0, and an NC below 2.0 was considered good [32, 48].

## Results

### Participants

Except a few cases, most of the data were collected by two researchers through interview from the community center users, and all the data collected through snowball sampling were self-measured by the respondents.

The average age of the respondents was 70.21 years (standard deviation [SD], 7.22 years), and 73.56% were female. More than three-quarters of the participants were not working because they were retired (37.02%) or stay-at-home moms (38.46%). The proportion of employed participants was 18.27%, those temporarily taking a leave of absence was 4.33%, and non-response was 1.92%.

### Measurement model

The NC of the measurement model in this study was 1.724 (NC = x^2^/df, x^2^ = 872.509, df = 506, *p* < .001). Although it is ideal when the *P* value of the model is not significant, it is likely to be influenced by the amount of data. Subsequently, after finding out that the overall fit indices of the measurement model in this study were good (RMSEA = .059, TLI = .921, CFI = .929, SRMR = .065), the research model was analyzed.

### Test of normality and convergent validity

The test of normality was necessary to obtain accurate results from the analysis using maximum likelihood. The range of the absolute skewness of each variable was between .013 and 2.360, which was considered acceptable [47, 48]. Although multivariate kurtosis violated normality assumption with the value of multivariate kurtosis of 99.015 and its critical ratio of 14.431, we decided to use all observed variables without transformation or elimination because the range of the absolute value of kurtosis in each variable was between .00 and 5.117, which was acceptable [49]. Furthermore, we used sub-scores of validated assessment tools, and all subcategories were considered necessary components for measuring each latent variable. Additionally, the total number of cases used in the analysis was more than the threshold for the application of the maximum likelihood method [32].

The mean and the SD of the variables and convergent validity of each latent variable are presented in Table 1. Except an observed variable of frequency, all latent variables used in the structural equation model were measured with proper items showing appropriate factor loadings (λ>.5) [32] (Table 1). Although the factor loading of frequency on participation was under .5, we used the item for analysis due to its essentiality as a component of the construct of participation.

**Table 1:** Confirmatory factor analysis (N = 208).

Latent variables	Observed variables (number of items)	Mean (SD)	Factor loading	*P*	Internal consistency
OB	Health (6)	2.59 (0.43)	.585	***	.716
	Identity (10)	2.45 (0.44)	.856	***	.605
	Relationship (16)	2.38 (0.49)	.719	***	.678
	Challenge and interest (19)	2.34 (0.47)	.835	***	.771
LeS	Psychological factor (4)	3.39 (0.60)	.907	***	.832
	Relaxational factor (4)	3.58 (0.58)	.920	***	.756
	Aesthetic factor (4)	3.35 (0.62)	.702	***	.855
	Social factor (4)	3.48 (0.60)	.876	***	.820
	Physiological factor (4)	3.56 (0.65)	.894	***	.871
	Educational factor (4)	3.43 (0.56)	.858	***	.827
Stress	Negative item 1 (1)	2.86 (0.69)	.687	***	
	Negative item 2 (1)	2.54 (0.73)	.571	***	
	Negative item 3 (1)	2.86 (0.78)	.724	***	
	Negative item 4 (1)	2.75 (0.74)	.579	***	
	Negative item 5 (1)	2.50 (0.85)	.731	***	
	Positive item 1 (1) ^†^	3.14 (0.73)	.143	*	
	Positive item 2 (1) ^†^	3.04 (0.73)	.231	**	
	Positive item 3 (1) ^†^	2.91 (0.69)	.388	***	
	Positive item 4 (1) ^†^	2.92 (0.78)	.517	***	
	Positive item 5 (1) ^†^	2.73 (0.72)	.412	***	
LiS	Item 1 (1)	3.01 (0.88)	.774	***	
	Item 2 (1)	3.00 (0.84)	.832	***	
	Item 3 (1)	3.16 (0.90)	.866	***	
	Item 4 (1)	2.91 (0.94)	.741	***	
	Item 5 (1)	2.63 (1.13)	.679	***	
SH	Cognition (2)	4.35 (0.77)	.840	***	.720
	Mobility (2)	4.20 (0.92)	.835	***	.792
	Self-care (2)	4.74 (0.57)	.720	***	.809
	Getting along (2)	4.62 (0.59)	.700	***	.736
	Life activities (2)	4.31 (0.75)	.875	***	.748
	Participation (2)	4.16 (0.88)	.856	***	.815
QOL	General QOL (1)	3.20 (0.81)	.847	***	
	Physical QOL (7)	3.24 (0.65)	.858	***	.829
	Psychological QOL (6)	3.23 (0.62)	.911	***	.824
	Social QOL (3)	3.09 (0.52)	.695	***	.646
	Environmental QOL (8)	3.24 (0.53)	.841	***	.826
Participation	Frequency (11)	30.39 (12.47)	.474	***	
	Restriction (11)	89.16 (16.67)	.738	***	
	Satisfaction (10)	58.65 (15.84)	.815	***	

***p < .001,

**p < .01,

*p < .05.

^† ^These five items were not used for analysis because of low factor loadings.

OB, occupational balance; LeS, leisure satisfaction; LiS, life satisfaction; SH, subjective health; QOL, quality of life.

### Correlation and discriminant validity

Table 2 presents the matrix of correlation between the latent variables. All seven latent variables were significantly associated with each other (Table 2). However, some coefficients were over .80, and a high level of correlation over .80 [47] may cause inadequate path coefficient or reliability problems because of multicollinearity [50].

**Table 2:** Matrix of correlation between latent variables (N = 208).

	OB	LeS	Stress	LiS	SH	QOL	Participation
OB	1						
LeS	.237**	1					
Stress	-.241**	-.477***	1				
LiS	.213*	.591***	-.632***	1			
SH	.211**	.474***	-.468***	.422***	1		
QOL	.282**	.632***	-.658***	.855***	.679***	1	
Participation	.249**	.748***	-.589***	.728***	.823***	.881***	1

OB, occupational balance; LeS, leisure satisfaction; LiS, life satisfaction; SH, subjective health; QOL, quality of life.

***p < .001,

**p < .01,

*p < .05.

To confirm that each latent variable was a distinct construct, discriminant validity was examined. We calculated the change in x^2^ between the unconstrained and the constrained model with limited covariance between the two variables. Discriminant validity was confirmed when Δx^2^ between the two models was higher than 3.84 [32]. Considering that all Δx^2^ between the two models were higher than 3.84 (change of x^2^ between quality of life and participation: 20.865, change of x^2^ between quality of life and life satisfaction: 46.542, change of x^2^ between subjective health and participation: 19.201), the latent variables of life satisfaction, subjective health, quality of life, and participation were confirmed to be distinct constructions. All four latent variables were inserted in the final model because we concluded that these four variables were distinctive, although they were highly associated with each other.

### Model modification

The fit indices of the research model were appropriate (NC = 1.788 [x^2^ = 908.136, df = 508], *p* < .001, RMSEA = .062, TLI = .914, CFI = .922, and SRMR = .0984). However, model modification was recommended because the model contained paths that were not statistically significant. We modified the research model to ensure statistically significant paths and improve fit indices. First, we removed direct paths that were not significant at the 95% confidence interval. Second, we added a new path or changed the direction of the path after confirmation with previous studies. A path from leisure satisfaction to stress [51–53] was added. The path from participation to subjective health was reversed [54].

The fit index of the final model was NC (x^2^/df) = 1.708 (x^2^ = 877.917, df = 514, *p* < .001). Overall indices were acceptable (RMSEA = .058, TLI = .923, CFI = .929, SRMR = .067). Except the direct path between occupational balance and stress (*p* = .060), all the direct paths in the modified model were significant at the 95% confidence interval. Although the path between occupational balance and stress was not statistically significant at the 95% confidence interval, we determined to retain the path in the final model because the association between occupational balance and stress was supported by previous studies, and we discovered that the overall fit indices decreased when the path was eliminated (NC = 1.712, *p* < .001, RMSEA = .059, TLI = .922, CFI = .929, SRMR = .071).

### Final model

**Fig 2:**
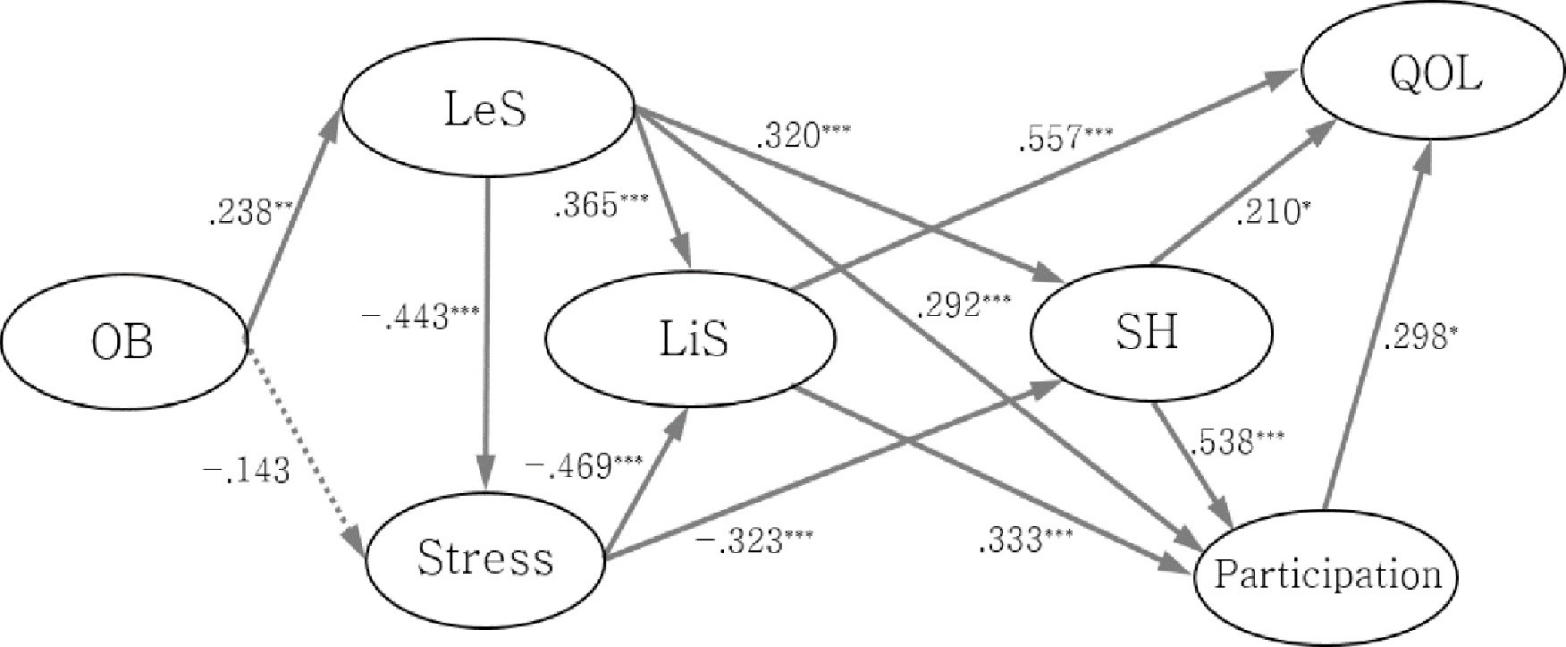
Final model. OB = occupational balance; LeS = leisure satisfaction; LiS = life satisfaction; SH = subjective health; QOL = quality of life. ***p < .001, **p < .01, *p < .05. Solid line demonstrates a significant path at 95% confidence interval. Dotted line demonstrates a significant path at 90% confidence interval.

Fig 2 illustrates the paths and coefficients in the final model. With the final model, we could not confirm the direct path between occupational balance and the four variables of life satisfaction, participation, subjective health, and quality of life. We found that leisure satisfaction and stress mediated the relationship between occupational balance and subjective health.

### Path analysis

The coefficient, standard error, and *p* value of paths in the final model are demonstrated in Table 3.

**Table 3:** Coefficient, standard error, and *p* value in the final model (N = 208).

Pathway	Non-standardized coefficient (B)	SE	Standardized coefficient (β)	CR	*P*
OB → LeS	.537	.177	.238	3.026	.002
OB → stress	-.274	.146	-.143	-1.878	.060
LeS → stress	-.375	.068	-.443	-5.519	***
LeS → SH	.387	.092	.320	4.212	***
LeS → LiS	.463	.088	.365	5.248	***
LeS → participation	6.206	1.385	.292	4.481	***
Stress → LiS	-.702	.123	-.469	-5.688	***
Stress → SH	-.462	.119	-.323	-3.891	***
LiS → QOL	.538	.085	.557	6.335	***
LiS → participation	5.577	1.123	.333	4.966	***
SH → QOL	.212	.098	.210	2.175	.030
SH → participation	9.432	1.198	.538	7.872	***
Participation → QOL	.017	.008	.298	2.136	.033

OB, occupational balance; LeS, leisure satisfaction; LiS, life satisfaction; SH, subjective health; QOL, quality of life; SE, standard error; CR, critical ratio.

***p < .001

Occupational balance had a direct effect on leisure satisfaction (β = .238, *p* = .002), and leisure satisfaction was a mediating factor between occupational balance and the four variables: stress, life satisfaction, subjective health, and participation. Additionally, occupational balance was directly associated with stress at the 90% confidence interval (β = -.143, *p* = .060).

Subjective health was directly associated with participation (β = .538, *p* < .001), stress (β = -.323, *p* < .001), leisure satisfaction (β = .320, *p* < .001), and quality of life (β = .210, *p* = .030). Quality of life was directly associated with life satisfaction (β = .557, *p* < .001) and participation (β = .298, *p* = .033).

### Direct and indirect effects analysis

The effects analysis among paths in the final model is presented in Table 4. To compare the size of effect, the effect of leisure satisfaction and stress, which was directly associated with occupational balance, was also illustrated along the effect of occupational balance. Occupational balance had a small- to medium-sized effect on the other variables [55]. Among occupational balance, leisure satisfaction, and stress, leisure satisfaction showed the largest effect followed by stress and occupational balance in terms of total effect on subjective health, quality of life, and participation.

**Table 4:** Direct and indirect effects of the study variables.

Exogenous variables	Endogenous variables	Direct effect (*p*)	Indirect effect (*p*)	Total effect (*p*)
OB	LeS	.238 (.002)		.238 (.005)
	Stress	-.143 (.060)	-.106 (.007)	-.249 (.007)
	LiS		.204 (.004)	.204 (.004)
	SH		.157 (.004)	.157 (.004)
	QOL		.212 (.004)	.212 (.004)
	Participation		.222 (.004)	.222 (.004)
LeS	Stress	-.443 (***)		-.443 (.004)
	LiS	.365 (***)	.208 (.004)	.573 (.004)
	SH	.320 (***)	.143 (.004)	.463 (.004)
	QOL		.634 (.004)	.634 (.004)
	Participation	.292(***)	.439 (.004)	.731 (.004)
Stress	LiS	-.469(***)		-.469 (.004)
	SH	-.323(***)		-.323 (.004)
	QOL		-.427 (.004)	-.427 (.004)
	Participation		-.330 (.004)	-.330 (.004)

OB, occupational balance; LeS, leisure satisfaction; LiS, life satisfaction; SH, subjective health; QOL, quality of life.

***p < .001.

## Discussion

To the best of our knowledge, this is the first study that confirms the effect of occupational balance on subjective health and quality of life using a structural equation modeling approach. Pathways in the final model explain the association between occupational balance and health-related factors. We found the value of occupational balance as an independent variable based on the results of the effects analysis. The findings support the possibility of using the concept of occupational balance for improving subjective health and quality of life for older adults. This could be applied to community-dwelling older adults, who are relatively healthy, as a preventative approach.

Gaining satisfaction is a requisite factor for achieving occupational balance [22] and essential for improving health [23, 25]. In previous studies about occupational balance, satisfaction-related variables were life satisfaction [1, 13, 22], leisure [13, 56], and stress [1, 2, 12, 22]. In the research model of this study, we hypothesized that the variables of life satisfaction, leisure satisfaction, and stress would mediate between occupational balance and both subjective health and quality of life. As we expected, leisure satisfaction and stress were the mediating factors in the final model. However, we could not find a direct association between occupational balance and life satisfaction. This might be because of the difference in time periods evaluated for occupational balance and life satisfaction. While life satisfaction was assessed with questions about satisfaction on overall life [41, 42], occupational balance was evaluated with questions about satisfaction experienced during the last four weeks [7, 33]. The occupational balance of the last four weeks might scarcely affect the entire life satisfaction of older adults in this cross-sectional study.

A weak association between occupational balance and stress may be caused by older adults’ coping strategies for stress [27]. Stress originating from excessive or rare participation in specific activities could be controlled applying the concept of occupational balance, directly modulating the level of involvement in related activities. While using the concept of occupational balance is an active way to relieve stress, older adults prefer reinterpreting or evading the stressful situation over solving the problem directly [7]. Another possibility is related to the level of occupational balance of the sample. A previous study [12] reported the association between a lower level of occupational balance and stress. However, the K-LBI score of the sample in this study indicated that they were maintaining some degree of occupational balance [7]. This might have affected the weak association between occupational balance and stress in this study. The distinctive structure between occupational imbalance and balance [3] can be another potential reason for the weak association between occupational balance and stress. Further study is recommended to collect definitive evidence of the association between occupational balance and stress.

We assumed that enhancing the level of occupational balance can lead to higher levels of leisure satisfaction or lower levels of stress based on the direct effect observed in the final model. Regarding leisure satisfaction and stress, they both had a direct influence on subjective health and an indirect influence on quality of life. Although the effect size was small, we may consider leisure activities or stress management as practical application methods to use the concept of occupational balance for improving older adults’ subjective health and quality of life. Therefore, it is necessary to measure individual levels of occupational balance to find target activities, and modulate their frequency to increase satisfaction or decrease stress of older adults. Subsequently, customized approaches should be followed to maintain the habit of modulated activity pattern. Community senior centers or public health centers are appropriate organizations that can adopt this type of approach to protect community-dwelling older adults from unhealthy daily activity patterns.

Currently, subjectivity is considered an important structure in the concept of occupational balance [1, 22]. However, objectivity also needs to be considered important in using the concept of occupational balance for protecting individuals’ health conditions. Since the score of occupational balance measurement is based on subjectivity, older adults may think that they are occupationally balanced when they are satisfied with their daily activity pattern, although the diversity, amount, or intensity of daily activities are inappropriate. Additional objective guidelines, such as professional counseling based on older adults’ physical and cognitive functions, need to be provided to reach an occupational balance that will help this population protect and improve their health and wellbeing.

The present study has some limitations. First, high correlations between the latent variables might affect the results despite all latent variables being measured using validated assessment tools and having confirmed discriminant validity. Second, the results of this study might disproportionately reflect the characteristics of female older adults because the proportion of female participants (73.56%) was higher than their proportion in the general Korean population (56.94%) [57]. Third, cultural differences between western countries and Korea may affect the results. Most of the studies we referred to were conducted in western countries. Due to the dearth of studies related to occupational balance, except one study [23], all previous studies addressing occupational balance were conducted in western countries. Considering that the concept of occupational balance is linked closely to daily pattern [22], mismatch may exist between the research model and the data in this study. Further comparison studies on occupational balance in different cultures or various age groups are needed to establish the effect of occupational balance on health-related variables. Additional variables related to health and wellbeing need to be investigated in future studies to clearly explain the influence of occupational balance on health and wellbeing. Furthermore, we suggest developing an intervention program based on occupational balance to confirm its practical effect on the life of older adults.

## Conclusion

The final model developed in this study shows the associations between occupational balance and subjective health, quality of life, and health-related variables. Higher levels of occupational balance have a positive effect on the aforementioned variables among relatively healthy older adults. These results support the relevance of using the concept of occupational balance to protect the health status and quality of life of older adults.

## Supporting information

S1 File Dataset. Minimal dataset of the study.
(SAV)
